# TIMP1 regulates ferroptosis in osteoblasts by inhibiting TFRC ubiquitination: an in vitro and in vivo study

**DOI:** 10.1186/s10020-024-01000-9

**Published:** 2024-11-23

**Authors:** Bo Peng, Zhiwei Feng, Ao Yang, Jinmin Liu, Jinwen He, Lihu Xu, Cong Tian, Xiaoyun Sheng, Yaobin Wang, Rongjin Chen, Xingwen Wang, Xiaojun Ren, Bin Geng, Yayi Xia

**Affiliations:** 1 Department of Orthopaedics, The Second Hospital of Lanzhou University, #82 Cuiyingmen, Lanzhou, Gansu, 730030 People’s Republic of China; 2 Intelligent Orthopedics Industry Technology Center of Gansu Province, Lanzhou, Gansu China; 3 Orthopaedic Clinical Research Center of Gansu Province, Lanzhou, Gansu China

**Keywords:** TIMP1, TFRC, Ubiquitination, Ferroptosis, Osteoblast, Iron metabolism

## Abstract

**Background:**

In clinical practice, alterations in the internal environment of type 2 diabetes can significantly affect bone quality. While the increased risk of fractures among diabetic patients is well-established, the precise mechanisms by which hyperglycemia influences bone quality remain largely unclear.

**Methods:**

Western blotting, immunohistochemistry (IHC), and micro-CT were used to examine ferroptosis-related protein expression and bone morphology changes in the bone tissues of type 2 diabetic mice. The CCK8 assay determined the optimal conditions for inducing ferroptosis in osteoblasts by high glucose and high fat (HGHF). Ferroptosis phenotypes in osteoblasts were analyzed using flow cytometry, Western blotting, and two-photon laser confocal microscopy. Transcriptomic sequencing of the control and HGHF groups, followed by bioinformatic analysis, identified and validated key genes. TIMP1 was knocked down in osteoblasts to assess its impact on ferroptosis, while TFRC expression was inhibited and activated to verify the role of TIMP1 in regulating ferroptosis through TFRC. The therapeutic effect of TIMP1 inhibition on osteoporosis was evaluated in a type 2 diabetic mouse model.

**Results:**

The expression of TIMP1 is increased in type 2 diabetic osteoporosis. In vitro, TIMP1 knockout inhibited ferroptosis in osteoblasts induced by high glucose and high fat (HGHF). However, overexpression of TFRC reversed the ferroptosis inhibition caused by TIMP1 knockout. Suppression of TIMP1 expression alleviated the progression of osteoporosis in type 2 diabetic mice. Mechanistic studies suggest that TIMP1 regulates HGHF-induced ferroptosis in osteoblasts through TFRC.

**Conclusion:**

This study demonstrates that TIMP1 expression is increased during type 2 diabetic osteoporosis and that TIMP1 promotes ferroptosis in osteoblasts by regulating TFRC. These findings suggest that TIMP1 is a promising novel therapeutic target for type 2 diabetic osteoporosis.

**Supplementary Information:**

The online version contains supplementary material available at 10.1186/s10020-024-01000-9.

## Introduction

Diabetic osteoporosis is a bone disease recognized as a leading cause of fractures, increased bone fragility, and reduced bone strength due to changes in bone microstructure and osteopenia in diabetic patients (Liu et al. 2022). Annually, over 9 million osteoporotic fractures occur globally, with a significant proportion linked to diabetic osteoporosis (Yang et al. 2022a, b), imposing a substantial burden on patients and healthcare systems. Recent research into the diabetic microenvironment and mineral homeostasis has identified distorted bone microarchitecture, imbalanced bone metabolism, and increased cortical porosity as hallmarks of diabetes-associated osteoporosis (Shanbhogue et al. 2017). Although clinical evidence indicates that the diabetic cellular microenvironment is detrimental to bone health, the precise pathophysiological and molecular mechanisms remain under investigation. It has been observed that osteoporosis induced by type 2 diabetes mellitus does not show a significant reduction in bone mineral density compared to type 1 diabetes mellitus (Napoli et al. 2017). Interestingly, type 2 diabetic osteoporosis is associated with more severe deterioration of bone microarchitecture and increased bone fragility (Khosla et al. 2021; Hofbauer et al. 2022). Therefore, the mechanisms underlying type 2 diabetic osteoporosis deserve further comprehensive investigation.

Osteoblasts, originating from bone marrow mesenchymal stem cells, are essential for bone formation, producing the extracellular matrix, depositing calcium, and expressing osteogenic factors to maintain bone tissue’s normal physiological functions (Zheng et al. 2020; Sheppard et al. 2022). In a diabetic environment, hyperglycemia impairs osteoblast number and function through various pathways, inhibiting bone formation and mineralization, thus contributing to bone damage (Bueno-Vargas et al. 2022; Xu et al. 2023).

Ferroptosis is a newly recognized form of cell death, characterized by iron-dependent lipid peroxidation, distinct from apoptosis, necrosis, and autophagy (Fang et al. 2021; Zheng et al. 2023; Ye et al. 2021). Emerging research has underscored a critical link between ferroptosis and glucose-lipid metabolism. Hyperglycemia and dyslipidemia, hallmarks of metabolic diseases like diabetes, foster oxidative stress and iron accumulation (Hofer et al. 2022; Yang et al. 2022a, b). Elevated glucose levels increase ROS production, which enhances lipid peroxidation, a critical step in ferroptosis (Wang et al. 2023a, b). Concurrently, dysregulated lipid metabolism, marked by elevated PUFAs and altered lipid handling, predisposes cells to ferroptosis (Sun et al. 2019), exacerbating cellular and tissue damage in metabolic disorders. Understanding these pathways opens avenues for therapeutic strategies targeting ferroptosis to mitigate metabolic disease progression and consequences. Some studies suggest that research on osteoblast ferroptosis could serve as a new avenue for combating type 2 diabetic osteoporosis (Jin et al. 2023). Zhang, Z, and Ma, H’s Studies demonstrated that osteoblast ferroptosis induced by a high-glucose and high-lipid microenvironment is closely associated with type 2 diabetes-induced osteoporosis (Zhang et al. 2022a, b; Ma et al. 2020).

TFRC has been shown to elevate intracellular iron levels by mediating iron uptake, thereby promoting ROS generation and lipid peroxidation ((Yi et al. 2022). TIMP1, a secretory protein primarily recognized for its inhibitory effect on matrix metalloproteinase 9 (MMP9), has not been studied for its mechanistic role in diabetic osteoporosis (Rao et al. 2022; Wu et al. 2022; Morrell et al. 2022; Yue et al. 2016). This study validates ferroptosis as a significant contributor to osteoblast death in diabetic osteoporosis, both in vitro and in vivo. We found that in a diabetic environment, TIMP1 initially binds to TFRC, leading to increased TFRC expression, thereby heightening osteoblast sensitivity to ferroptosis. Furthermore, targeting ferroptosis significantly reduces osteoblast mortality and trabecular bone degeneration. Our findings provide a comprehensive understanding of the pathways contributing to diabetic osteoporosis and suggest potential targets for future therapeutic interventions.

## Materials and methods

### Animals

Male C57BL/6 mice (6–8 weeks old, 18 ± 2 g) were housed under standard SPF conditions at the Lanzhou Animal Research Institute, Chinese Academy of Agricultural Sciences, for 12 weeks. The facility maintained pathogen-free conditions, including the absence of zoonotic infections, major infectious pathogens, and other agents that could significantly affect the animals or the study. Environmental parameters were controlled at 20–25 °C, with 55–60% humidity and a 12-hour light/dark cycle. Mice were provided with a high-fat diet (HFD) and water ad libitum. In the fifth week, a low dose of streptozotocin (STZ) was administered over 4 days to establish the type 2 diabetic osteoporosis. Blood glucose and body weight were measured biweekly, and fasting insulin levels were recorded at euthanasia.

Male C57BL/6 mice (6–8 weeks old) were randomly assigned to three groups: LV-NC, LV-NC + STZ & HFD, and LV-shTIMP1 + STZ & HFD. The LV-NC group received no special dietary intervention, while the LV-NC + STZ & HFD and LV-shTIMP1 + STZ & HFD groups were subjected to low-dose STZ combined with HFD. Starting from week 5, mice in all groups received weekly injections of 5 × 10⁷ TU lentivirus (LV-NC or LV-shTIMP1) at the distal femur surface for 8 consecutive weeks. Mice were euthanized 7 days after the final lentivirus injection.

### Cell culture

The MC3T3-E1 cell line, derived from mice, was obtained from the Cell Bank of Peking Union Medical College, Beijing, China. Cells were cultured in α-Minimum Essential Medium (α-MEM) supplemented with 10% fetal bovine serum (Hyclone, UT, USA) and maintained at 37 °C in a humidified atmosphere with 5% CO_2_. To simulate high glucose and high fat (HGHF) conditions, MC3T3-E1 cells were cultured in osteogenic α-MEM with the addition of 25.5 mM glucose and free fatty acids (palmitic acid and oleic acid in a 1:2 ratio). Cells were exposed to varying concentrations of free fatty acids for 12, 24, and 48 h to determine optimal conditions. Following this, the medium was supplemented with ferrostatin-1 (Fer-1, 10 µM), an inhibitor of ferroptosis; 3-methyladenine (3-MA, 5 µM), an autophagy inhibitor; Z-VAD-FMK (10 µM), an apoptosis inhibitor; and necrostatin-1 (Nec-1, 50 µM), a necrosis inhibitor, to elucidate the effects of HGHF on osteoblasts.

### Cell counting kit-8 assay

The cells were distributed at a density of 2 × 10^3^ cells per well in a 96-well plate. After various treatments, 90 µL of complete medium was introduced into each well, followed by the addition of 10 µL of CCK-8 reagent (Biosharp, China). The absorbance at 450 nm was measured using a microplate reader (Power Wave XS2, USA) after incubating for 2 h. Each experimental group was established with a minimum of 3 duplicate wells.

### Immunofluorescence (IF)

Cells seeded on glass-bottom dishes (Biosharp) were fixed with 4% paraformaldehyde for 20 min, permeabilized with 0.1% Triton X-100 for 10 min, and then washed with PBS. Blocking was performed with 5% goat serum for 1 h. Primary antibodies anti-TIMP1 (Abmart, TA7007) (dilution: 1:100) and anti-TFRC (SANTA CRUZ, sc-393719) (dilution: 1:150) were added and incubated overnight at 4 °C. The next day, cells were incubated with secondary antibodies: goat anti-rabbit IgG (1:300; AFFINITY, S0006) and goat anti-mouse IgG (1:300; Proteintech, SA00014-10) in the dark for 1 h. Nuclei were stained with DAPI (Biosharp). Imaging was performed using a two-photon laser confocal microscope (Carl Zeiss, Zeiss LSM880).

### Micro-computed tomography (Micro-CT) analysis

Micro-CT imaging was performed using a Skyscan 1172 system (Skyscan, Kontich, Belgium), with a spatial resolution of 12 μm and an X-ray source set to 80 kV / 100 mA. Volume reconstruction and data analysis were conducted using NRecon software (version 1.6). Ex vivo scans of the distal femora from each mouse cohort were obtained with a SkyScan-1176 micro-CT scanner (Bruker MicroCT, Belgium), also at a spatial resolution of 12 μm. The reconstructed images were processed with NRecon (version 1.6), and subsequent analyses were carried out using CTAn software (version 1.8). To assess the microarchitecture of the distal femur, metrics including trabecular bone volume fraction (BV/TV, %), trabecular number (Tb.N, /mm), trabecular thickness (Tb.Th, mm), and trabecular separation (Tb.Sp, mm) were quantified. Additionally, bone mineral density (BMD, g/cm³) was measured.

### H&E staining

Mouse femora were fixed in 4% paraformaldehyde (PFA) for 1 day, followed by decalcification in 10% ethylenediaminetetraacetic acid (EDTA) for 21 days. The specimens were embedded in paraffin and sectioned at a thickness of 4 μm. Cellular staining was performed using an H&E staining kit (Solarbio), and the sections were subsequently mounted. The mouse femora were then observed under a light microscope (Olympus, Tokyo, Japan).

### Immunohistochemical (IHC) analysis

Mouse femur samples were fixed in 4% paraformaldehyde for one day, decalcified in 10% EDTA for 25 days, and then embedded in paraffin. Tissue sections were cut at a thickness of 4 μm. The slides were deparaffinized, dehydrated, rehydrated, and then subjected to antigen blocking and retrieval. Primary antibodies, including anti-TIMP1 (Immunoway, T4658, 1:50), anti-TFRC (Proteintech, 65236-1-Ig, 1:100), anti-ACSL4 (Proteintech, 22401-1-AP, 1:100), anti-GPX4 (Affinity, DF6701, 1:100), and anti-PTGS2 (Proteintech, 66351-1-Ig, 1:100), were applied to the slides and incubated overnight. After washing, the corresponding secondary antibodies were incubated for 30 min.

### Western blot

Proteins were extracted from osteoblasts or bone tissue using RIPA buffer (Beyotime, China) and quantified using a BCA protein assay kit (Beyotime). The proteins were then mixed with loading buffer at a 1:3 ratio and boiled for 10 min. Following separation by SDS-PAGE, the proteins were transferred onto PVDF membranes (Millipore, USA). The membranes were blocked with 5% BSA at 4 °C for 1 h and incubated overnight at 4 °C with the following primary antibodies: anti-TIMP1 (Cell Signaling Technology, 63363), anti-TFRC (Abcam, AB214039), anti-PTGS2 (Proteintech, 16837-1-AP), anti-GPX4 (Abmart, T56959), and anti-β-actin (Proteintech, 10494-1-AP). After primary antibody incubation, the membranes were treated with horseradish peroxidase-coupled secondary antibodies (Proteintech, 1:5000) for 1 h. Finally, signal bands were visualized using an ECL kit (Biosharp, China), and their grey values were analyzed using ImageJ software (Bethesda, MD, USA).

### Quantitative real-time PCR

The extraction of RNA was performed using TRIzol reagent. Afterwards, the process of cDNA synthesis was carried out using the PrimeScript^®^ RT Master Mix Kit (TaKaRa, Japan). Quantitative real-time PCR (QRT-PCR) was conducted using the LightCycler 480 PCR instrument (Roche, USA). The expression values were standardized using the internal control β-Actin. The 2^-ΔΔCt^ method was employed to ascertain the RNA levels. The primer sequences can be found in Supplementary.

### Transcriptome sequencing

Total RNA was extracted from osteoblasts treated or untreated with HGHF using TRIzol reagent (Invitrogen, CA, USA). RNA concentration and purity were assessed using a spectrophotometer (Thermo Fisher Nanodrop 2000, USA). Each sample provided 2.5 ng of RNA for library preparation. RNA was fragmented by heating to 95 °C, with removal of 3′-phosphate groups. Sequencing adapters were then ligated to the 3′ and 5′ ends. Libraries were constructed via reverse transcription and PCR amplification. The PCR products were purified with the AMPure XP system, and library quality was evaluated using the Agilent Bioanalyzer 2100. The libraries were sequenced and analyzed on the Illumina platform.

### Cell transfection

We employed specific shRNA and lentivirus, custom-synthesized by GeneChem Co., Ltd (Shanghai, China), to achieve TIMP1 knockdown. For TFRC, knockdown and overexpression were carried out via transient transfection using siRNA and plasmids respectively, both of which were custom-made by GenePharma Co., Ltd. (Shanghai, China). Detailed sequences of the shRNA, oeRNA, and siRNA can be found in the supplementary materials.

### Lipid peroxidation measurement

Changes in lipid peroxidation were assessed using the C11 BODIPY 581/591 lipid peroxidation fluorescent probe (MX5211, MKbio, China). Cells were washed with PBS and then incubated with C11-BODIPY 581/591 (final concentration 2 µM) at 37 °C for 60 min. Subsequently, the stained cells were collected, centrifuged at 4 °C (300×g) for 10 min, washed, and resuspended in PBS for flow cytometry analysis. Detection was performed using a flow cytometer (Beckman). At least 10,000 cells were recorded per read.

### ROS measurement

Intracellular ROS levels were measured using a Reactive Oxygen Species Detection Kit (Shanghai Life-iLab Biotech Co., Ltd). Cells were seeded in six-well plates and allowed to adhere before undergoing various treatments. After treatment, cells were collected and washed. The membrane-permeable fluorescent probe 2′,7′-dichlorofluorescein (DCFH-DA) was incubated at a dilution of 1:1000 for 30 min. After washing with serum-free medium, cells were sorted by flow cytometry. Intracellular ROS levels were expressed as the mean fluorescence intensity.

### MDA measurement

Intracellular MDA levels were measured using a Cell Lipid Peroxidation MDA Assay Kit (A003-4-1, Nanjing Jiancheng Bioengineering Institute, Jiangsu, China). After the specified treatments, osteoblasts were harvested and lysed in RIPA buffer. The cell lysates were centrifuged at 12,000 × g for 5 min, and the supernatant was collected for subsequent experiments. The MDA assay was performed strictly according to the manufacturer’s instructions. Protein concentration, required for the final calculation of MDA levels, was determined using a BCA Protein Assay Kit (Beyotime).

### Fe²⁺detection

Intracellular Fe²⁺ levels were assessed using the FerroOrange ferrous ion fluorescent probe (MX4559-24UG, MKBio). Cultured cells were subjected to specific treatments, and the FerroOrange fluorescent probe was added to the cells at a final concentration of 1 µM, followed by incubation at 37 °C for 30 min. Fe²⁺ levels were then detected using confocal microscopy and flow cytometry. The procedure was carried out strictly according to the manufacturer’s instructions.

### Co-immunoprecipitation (Co-IP)

After three PBS washes, cells were lysed with RIPA lysis buffer for 30 min at 4 °C as per the manufacturer’s instructions. The cells were then carefully scraped, collected, and centrifuged at 14,000 rpm for 15 min at 4 °C to obtain the supernatant. Protein samples were then incubated with either species-specific IgG or the corresponding primary antibody, along with protein A/G agarose beads, and rotated slowly overnight at 4 °C on a rotary shaker. The magnetic bead-antibody complexes were washed with a washing solution. Finally, 1× SDS-PAGE loading buffer was added, the beads were resuspended, and the samples were boiled for 8 min before performing protein electrophoresis.

### Ubiquitination measurement

MC3T3-E1 cells (with or without HGHF treatment) were processed using the Protein A/G Immunoprecipitation Kit (BeaverBeads^®^, 22202-20). After lysing the osteoblasts, a portion of the cell lysate was used for input analysis, with the remainder set aside. Protein A/G PLUS-Agarose was pre-cleared at 4 °C for 1 h. The supernatant was then incubated with a TIMP1 antibody (SANTA CRUZ, sc21734) on a rotating device at room temperature for 15 min, followed by overnight incubation with Protein A/G PLUS-Agarose on a rotating device at 4 °C to collect the immunoprecipitated proteins. The next day, the immunoprecipitated proteins were washed three times with PBS and boiled in 1× loading buffer for 5 min. Western blotting was performed using antibodies against Ubiquitin (Cell Signaling Technology, 20326), TIMP1 (Cell Signaling Technology, 63363), and TFRC (Abcam, AB214039).

### Statistical analysis

Data analysis was performed using GraphPad Prism 8 software. Statistical results are presented as the mean ± standard error of the mean (SEM) of three independent measurements. Independent sample t-tests or analysis of variance (ANOVA) were employed in this study. A p-value of < 0.05 was considered to indicate a statistically significant difference.

## Results

### Diabetes-induced ferroptosis in osteoblasts and osteoporosis

**Fig. 1:**
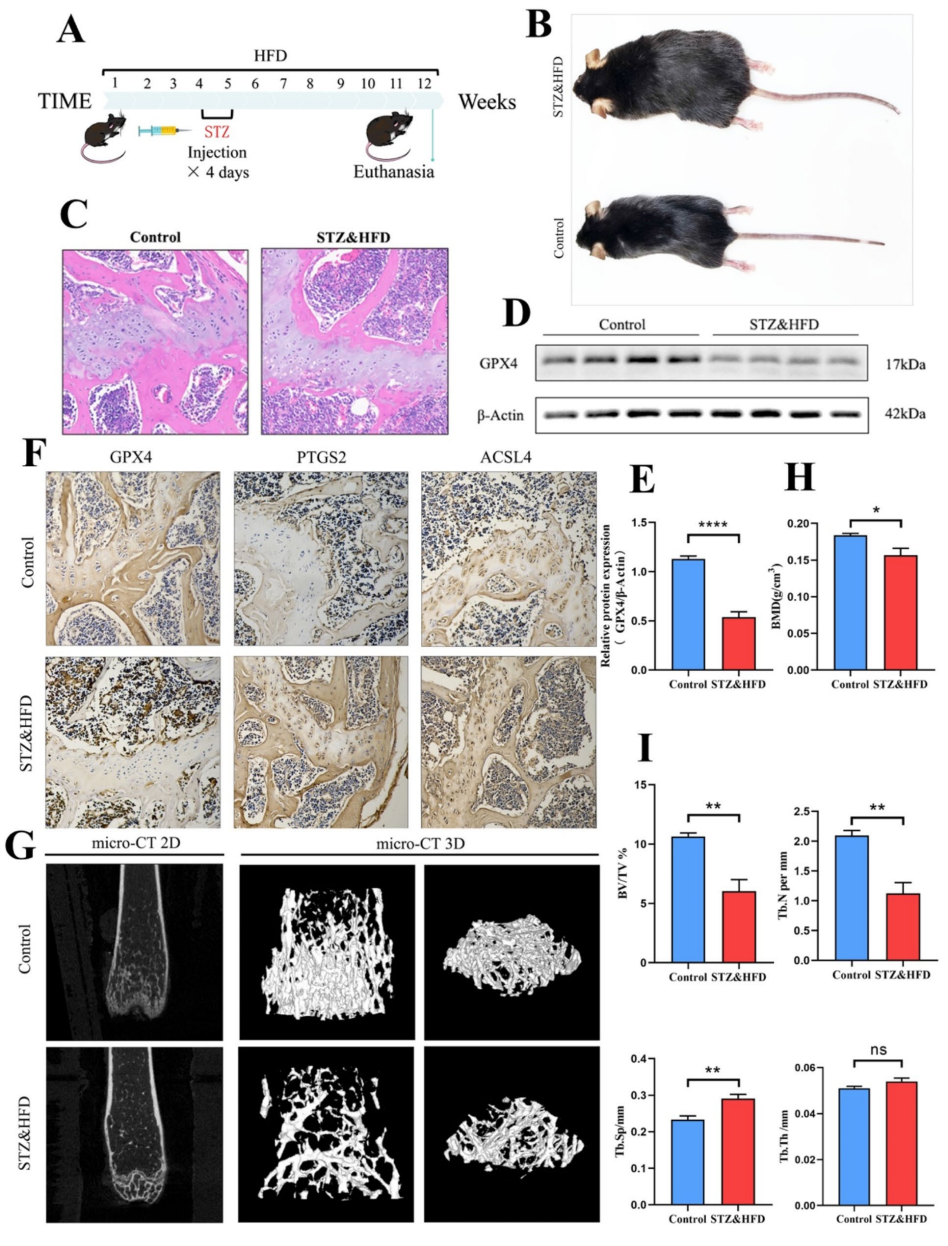
Diabetes-induced ferroptosis in osteoblasts and osteoporosis (**A**) Schematic representation of the diabetic osteoporosis mouse model induced by low-dose STZ injection in HFD-fed mice. (**B**) Gross images of control and STZ&HFD mice. (**C**) Histological images of distal femoral tissue sections stained with H&E. (**D**) Western blot (WB) analysis of GPX4 expression levels in femoral bone tissue (*n* = 4). (**E**) GPX4 expression analysis based on WB results. (**F**) Immunohistochemical images showing GPX4, PTGS2, and ACSL4 expression in control and STZ&HFD mice (*n* = 4). (**G**) 2D and 3D micro-CT images of the distal femur in control and STZ&HFD mice. (**H**) Quantitative analysis of trabecular bone density (BMD) at the distal femur in control and STZ&HFD mice (*n* = 4). (**I**) Quantitative analysis of BV/TV, Tb.Th, Tb.Sp, and Tb.N in the trabecular bone of the distal femur in control and STZ&HFD mice (*n* = 4). Data are presented as mean ± SEM (*P < 0.05, **P < 0.01, ***P < 0.001, ns, no statistical significance)

Firstly, a diabetic osteoporosis mouse model was established using a high-fat diet (HFD) (Fig. 1A) and low-dose streptozotocin (STZ) injection (Fig. 1B). Histological examination, including H&E staining and micro-CT analysis, revealed alterations in bone morphology. Specifically, H&E staining showed a reduction in trabecular surface area and number in the STZ&HFD mice (Fig. 1C). Micro-CT imaging data indicated a significant reduction in bone volume in the STZ&HFD mice compared to the control group, with notable changes in various bone parameters: decreases in bone mineral density (BMD), bone volume to total volume (BV/TV), and trabecular number (Tb.N), an increase in trabecular separation (Tb.Sp), but no significant change in trabecular thickness (Tb.Th) (Fig. 1G-I). Additionally, Western blot analysis revealed downregulation of the key ferroptosis protein GPX4 in the bone structure of the STZ&HFD mice (Fig. 1D, E). Immunohistochemical analysis showed decreased GPX4 expression, while PTGS2 and ACSL4 expressions were increased (Fig. 1F). These results collectively suggest a link between osteoblast ferroptosis and impaired bone structure in the STZ&HFD mouse model.

### HGHF-induced ferroptosis in osteoblasts

**Fig. 2:**
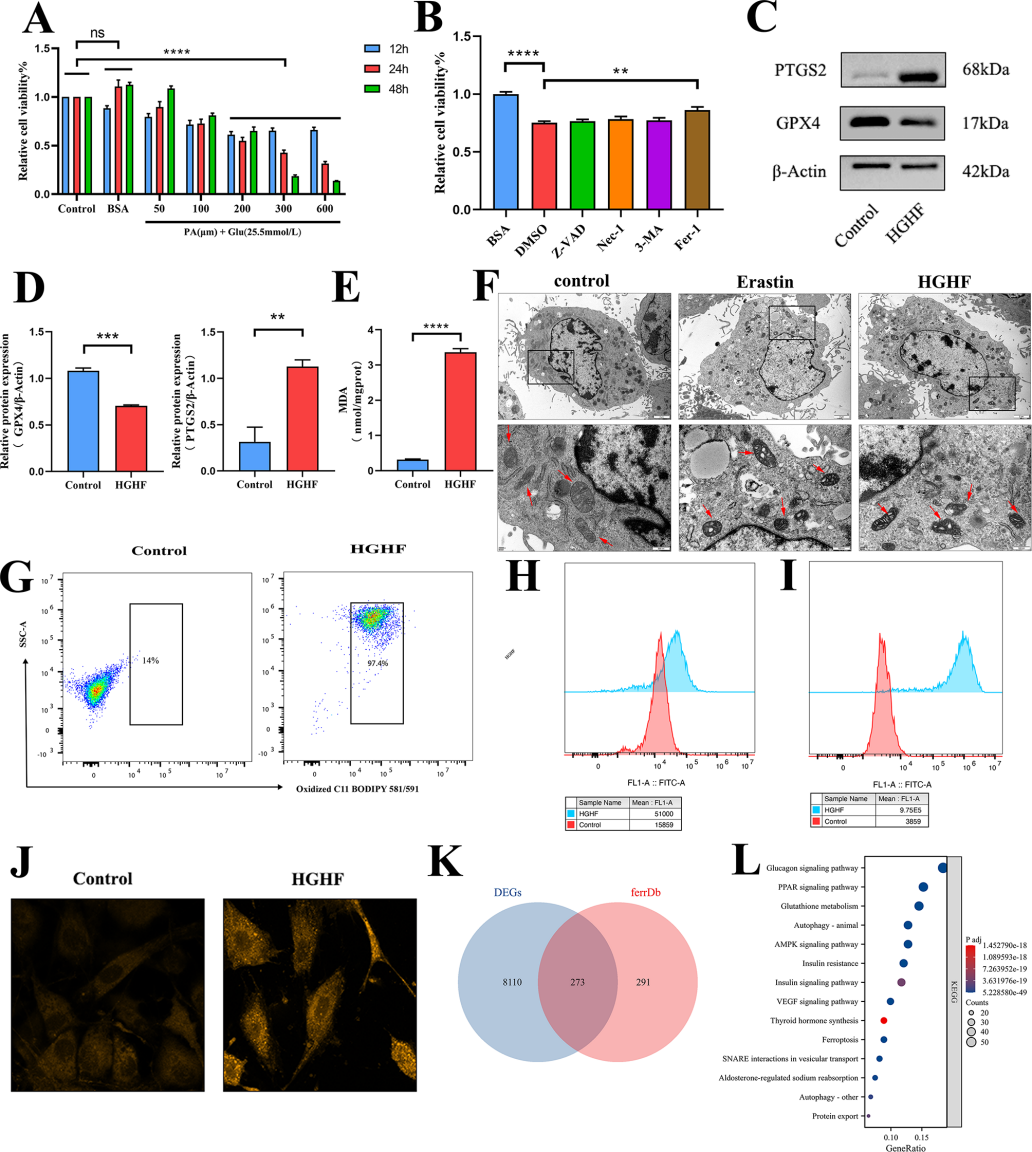
HGHF-induced ferroptosis in osteoblasts in vitro (**A**) Osteoblasts were treated with BSA (5.5 mmol•L^ − 1^ glucose) or various concentrations of PA and twice the concentration of OA (25.5 mmol•L ^− 1^ glucose) for different durations, followed by CCK-8 assay assessment. (**B**) CCK-8 assay of osteoblasts treated with HGHF containing BSA, DMSO (solvent), Z-VAD-FMK (apoptosis inhibitor), Fer-1 (ferroptosis inhibitor), Nec-1 (necrosis inhibitor), or 3-MA (autophagy inhibitor) for 24 h. (**C**) GPX4 and PTGS2 expression levels in control and HGHF-treated osteoblasts. (**D**) WB analysis of GPX4 and PTGS2 expression in osteoblasts (normalized to β-Actin control band intensity, *n* = 3). (**E**) Quantitative measurement of MDA levels in osteoblasts using an MDA assay kit. (**F**) Representative transmission electron microscopy images of control, Erastin, and HGHF-treated osteoblasts. (**G**) BODIPY 581/591 C11 probe estimation of lipid peroxidation levels in osteoblasts (assessed by flow cytometry). (**H**) Flow cytometric estimation of ROS levels in osteoblasts. (**I**) FerroOrange staining to estimate intracellular Fe^2+^ levels (assessed by flow cytometry). (**J**) Two-photon laser confocal microscopy images showing Fe^2+^ levels in osteoblasts after FerroOrange staining. (**K**) Differentially expressed genes in RNA-sequencing of control and HGHF-treated osteoblasts intersected with ferroptosis-related genes. (**L**) KEGG enrichment analysis of some differentially expressed genes in RNA-sequencing of control and HGHF-treated osteoblasts. Data are presented as mean ± SEM (*P < 0.05, **P < 0.01, ***P < 0.001, ns, no statistical significance)

Osteoblasts in the HGHF group were exposed to a glucose concentration of 25.5 mmol•L ^− 1^, simulating a diabetic environment, while the control group was treated with 5.5 mmol•L ^− 1^ glucose. Various concentrations of palmitic acid (PA) were tested to determine the optimal induction conditions, maintaining a PA to oleic acid (OA) ratio of 1:2 (Fig. 2A). The results demonstrated that HGHF treatment reduced osteoblast viability in a time- and dose-dependent manner. When the PA concentration exceeded 200 µmol•L^ − 1^, a significant decrease in cell viability was observed compared to the control group. At higher PA concentrations and prolonged incubation times, a substantial number of osteoblasts detached and floated, as observed under a light microscope, making it challenging to collect sufficient cells for subsequent experiments. Consequently, in this study, osteoblasts were treated with 300 µmol•L^ − 1^ PA and 600 µmol•L ^− 1^ OA for 24 h to induce cellular responses for further analyses. In line with previous reports (Ma et al. 2020), we explored the relative contributions of different forms of programmed cell death to HGHF-induced osteoblast death using a CCK-8 assay to evaluate the protective effects of various cell death inhibitors (Fig. 2B). The results indicated that only the ferroptosis-specific inhibitor, Fer-1, significantly prevented HGHF-induced osteoblast death, while other inhibitors had negligible effects. Western blot analysis corroborated these findings, revealing a reduction in GPX4 protein levels and an increase in PTGS2 protein expression in osteoblasts exposed to HGHF (Fig. 2C, D). To evaluate intracellular lipid peroxidation, cells were incubated with the C11-BODIPY fluorescent probe, and lipid peroxidation levels were quantified via flow cytometry. The data demonstrated a marked increase in lipid peroxidation following HGHF stimulation (Fig. 2G). Malondialdehyde (MDA), a byproduct of lipid peroxidation, exhibited a similar pattern (Fig. 2E). Transmission electron microscopy captured mitochondrial morphology in the control, HGHF, and positive control groups treated with Erastin. Mitochondria in both the HGHF and Erastin groups exhibited characteristic morphological alterations, including increased density, reduced volume, and diminished cristae (Fig. 2F). Furthermore, a substantial rise in reactive oxygen species (ROS) levels was detected in the HGHF group compared to untreated osteoblasts (Fig. 2H). To verify intracellular Fe²⁺ levels, the specific probe FerroOrange was employed, with detection performed via both flow cytometry and two-photon laser confocal microscopy (Fig. 2I, J). In all cases, a pronounced increase in Fe²⁺ levels was observed in osteoblasts treated with HGHF. Transcriptomic sequencing was conducted on osteoblasts with and without HGHF treatment. Differentially expressed genes (DEGs) were filtered with a p-value < 0.05 and intersected with ferroptosis-related genes from FerrDb (Database URL: http://www.zhounan.org/ferrdb)., identifying a total of 273 ferroptosis-related DEGs (Fig. 2K). Moreover, Kyoto Encyclopedia of Genes and Genomes (KEGG) pathway enrichment analysis of the sequencing data revealed significant alterations in the ferroptosis pathway (Fig. 2L).

### TFRC increases the sensitivity of osteoblasts to ferroptosis

**Fig. 3:**
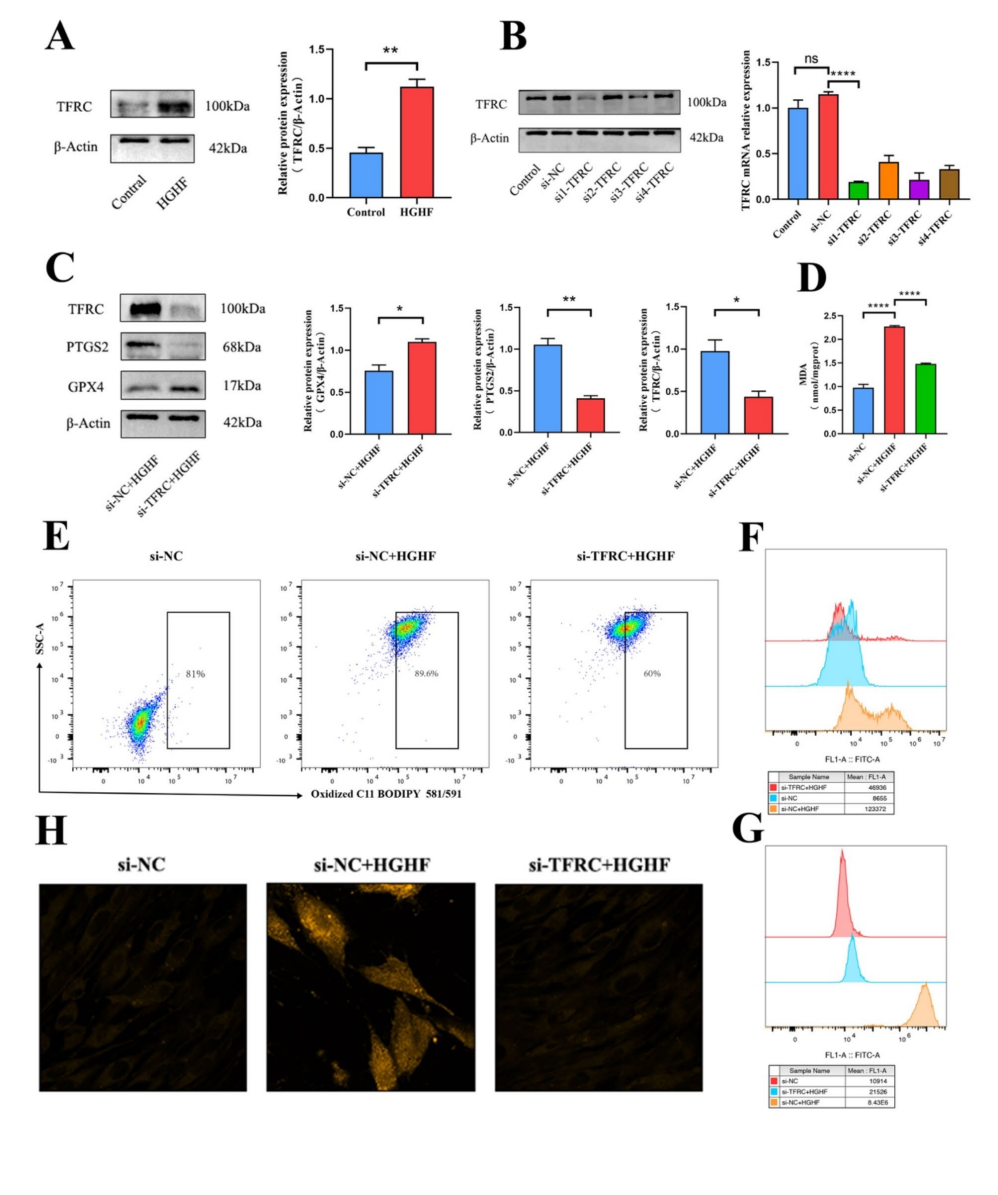
TFRC increases the sensitivity of osteoblasts to ferroptosis in vitro (**A**) TFRC expression in Control and HGHF osteoblasts, and TFRC expression analysis based on WB analysis (*n* = 3). (**B**) qRT-PCR and WB analysis of TFRC mRNA and protein expression levels in si-TFRC and si-NC groups. (**C**) WB analysis of PTGS2, GPX4, and TFRC protein expression levels, with WB result analysis (normalized to β-Actin control band intensity, *n* = 3). (**D**) Quantitative measurement of MDA levels in osteoblasts using an MDA assay kit. (**E**) BODIPY 581/591 C11 probe estimation of lipid peroxidation levels in osteoblasts (assessed by flow cytometry). (**F**) Flow cytometric estimation of ROS levels in osteoblasts. (**G**) FerroOrange staining to estimate intracellular Fe^2+^ levels (assessed by flow cytometry). (**H**) Two-photon laser confocal microscopy images showing Fe^2+^ levels in osteoblasts after FerroOrange staining. Data are presented as mean ± SEM (*P < 0.05, **P < 0.01, ***P < 0.001, ns, no statistical significance)

Western blot analysis revealed a significant upregulation of TFRC protein expression in osteoblasts treated with HGHF (Fig. 3A). To elucidate the role of TFRC in osteoblasts, siRNAs were utilized to knock down TFRC mRNA and protein levels (Fig. 3B). Western blot results indicated that, compared to the si-NC group, the si-TFRC group displayed increased GPX4 expression and decreased PTGS2 expression following exposure to HGHF-containing medium (Fig. 3C). MDA levels, assessed by zymography, were significantly lower in si-TFRC osteoblasts (Fig. 3D). These findings suggest that ferroptosis was mitigated in the si-TFRC group. Flow cytometry further confirmed changes in ferroptosis-associated markers. Specifically, following HGHF stimulation, the si-TFRC group exhibited reduced levels of ROS, Fe²⁺, and lipid peroxidation (Fig. 3E-G). Two-photon laser confocal microscopy corroborated these results, showing consistently lower intracellular Fe²⁺ levels (Fig. 3H). Collectively, these data demonstrate that TFRC enhances osteoblast sensitivity to HGHF-induced ferroptosis.

### TIMP1 is involved in the regulation of ferroptosis in osteoblasts

**Fig. 4:**
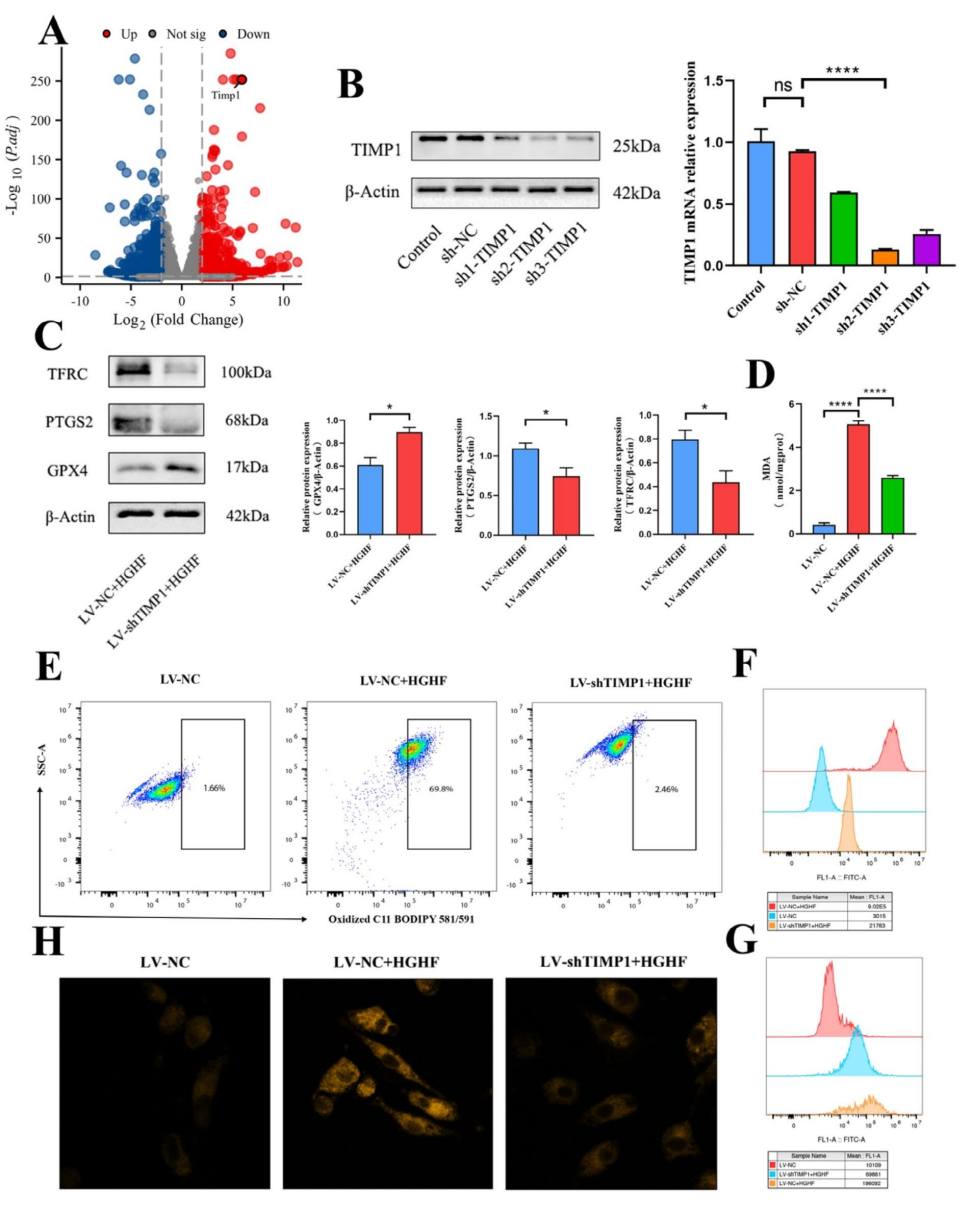
TIMP1 is involved in the regulation of ferroptosis in osteoblasts in vitro. (**A**) Bioinformatics analysis of differentially expressed genes in RNA-sequencing of control and HGHF-treated osteoblasts. (**B**) qRT-PCR and WB analysis of TIMP1 mRNA and protein expression levels in LV-shTIMP1 and LV-NC groups. (**C**) WB analysis of PTGS2, GPX4, and TFRC protein expression levels, with WB result analysis (normalized to β-Actin control band intensity, *n* = 3). (**D**) Quantitative measurement of MDA levels in osteoblasts using an MDA assay kit. (**E**) BODIPY 581/591 C11 probe estimation of lipid peroxidation levels in osteoblasts (assessed by flow cytometry). (**F**) FerroOrange staining to estimate intracellular Fe^2+^ levels (assessed by flow cytometry). (**G**) Flow cytometric estimation of ROS levels in osteoblasts. (**H**) Two-photon laser confocal microscopy images showing Fe^2+^ levels in osteoblasts after FerroOrange staining. Data are presented as mean ± SEM (*P < 0.05, **P < 0.01, ***P < 0.001, ns, no statistical significance)

Transcriptomic sequencing data revealed significant differential expression of the ferroptosis-related gene TIMP1 in osteoblasts treated with HGHF (Fig. 4A). To investigate the role of TIMP1 in osteoblast function, a lentiviral-mediated knockout strategy was employed. Following selection with 10 µg/mL puromycin, both TIMP1 mRNA and protein levels were significantly reduced (Fig. 4B). Functional analyses demonstrated that TIMP1 knockdown markedly suppressed ferroptosis in HGHF-treated osteoblasts. Flow cytometry analysis revealed notable reductions in ROS, lipid peroxidation, and Fe²⁺ levels in the LV-shTIMP1 group. Specifically, after HGHF stimulation, the LV-shTIMP1 group displayed significantly lower levels of ROS, Fe²⁺, MDA, and lipid peroxidation compared to the LV-NC group (Fig. 4E-G). Two-photon laser confocal microscopy corroborated these results, showing similarly reduced intracellular Fe²⁺ levels (Fig. 4H). Additionally, MDA assays confirmed that TIMP1 knockdown suppressed MDA production in osteoblasts (Fig. 4D). Consistent with these findings, Western blot analysis further demonstrated that TIMP1 knockout alleviated the decrease in GPX4 protein levels and the increase in PTGS2 protein levels typically associated with ferroptosis (Fig. 4C). Notably, despite exposure to HGHF, TFRC expression levels did not increase as expected in the LV-shTIMP1 group. In conclusion, these results indicate that TIMP1 knockout significantly inhibits both the abnormal upregulation of TFRC and the induction of ferroptosis in osteoblasts.

### TIMP1 regulates ferroptosis in osteoblasts via TFRC

**Fig. 5:**
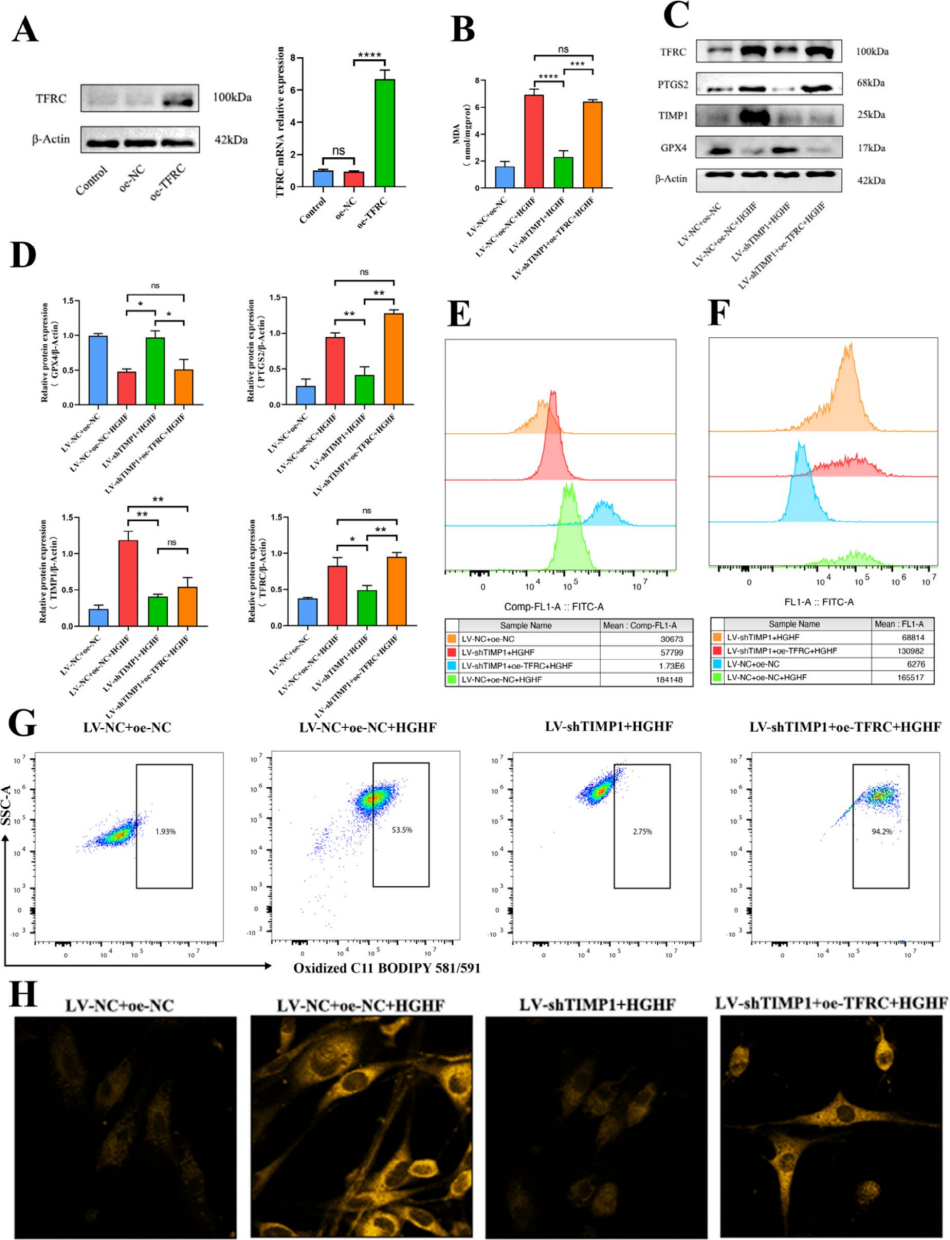
TIMP1 Regulates Osteoblast Ferroptosis via TFRC. (**A**) qRT-PCR and WB analysis of TFRC mRNA and protein expression levels in oe-TFRC and oe-NC groups. (**B**) Quantitative measurement of MDA levels in osteoblasts using an MDA assay kit. (**C**) WB analysis of PTGS2, GPX4, TFRC, and TIMP1 protein expression levels. (**D**) WB result analysis (normalized to β-Actin control band intensity, *n* = 3). (**E**) FerroOrange staining to estimate intracellular Fe^2+^ levels (assessed by flow cytometry). (**F**) Flow cytometric estimation of ROS levels in osteoblasts. (**G**) BODIPY 581/591 C11 probe estimation of lipid peroxidation levels in osteoblasts (assessed by flow cytometry). (**H**) Two-photon laser confocal microscopy images showing Fe^2+^ levels in osteoblasts after FerroOrange staining. Data are presented as mean ± SEM (*P < 0.05, **P < 0.01, ***P < 0.001, ns, no statistical significance)

TFRC overexpression was achieved through plasmid vectors, leading to a significant increase in both TFRC mRNA and protein levels (Fig. 5A). Osteoblasts with lentiviral-mediated TIMP1 knockdown (LV-shTIMP1) were subsequently transfected to overexpress TFRC. Flow cytometry analysis of ROS, lipid peroxidation, and intracellular Fe²⁺ levels revealed that TFRC overexpression consistently reversed the effects of TIMP1 knockdown on osteoblasts (Fig. 5E-G). This reversal was further supported by two-photon laser confocal microscopy, which confirmed the restoration of intracellular Fe²⁺ levels (Fig. 5H). MDA assay results showed no significant difference between the LV-shTIMP1 + oe-TFRC group and the LV-NC + oe-NC group following HGHF treatment (Fig. 5B). Western blot analysis demonstrated that, similar to the LV-shTIMP1 group, the LV-shTIMP1 + oe-TFRC group displayed reduced TIMP1 protein levels. However, the LV-shTIMP1 + oe-TFRC group exhibited even lower GPX4 levels and higher PTGS2 levels (Fig. 5C, D). These findings support the hypothesis that TFRC functions as a downstream effector of TIMP1, directly modulating ferroptosis in osteoblasts.

### TIMP1 influences ferroptosis in osteoblasts by regulating the stability of TFRC protein

**Fig. 6:**
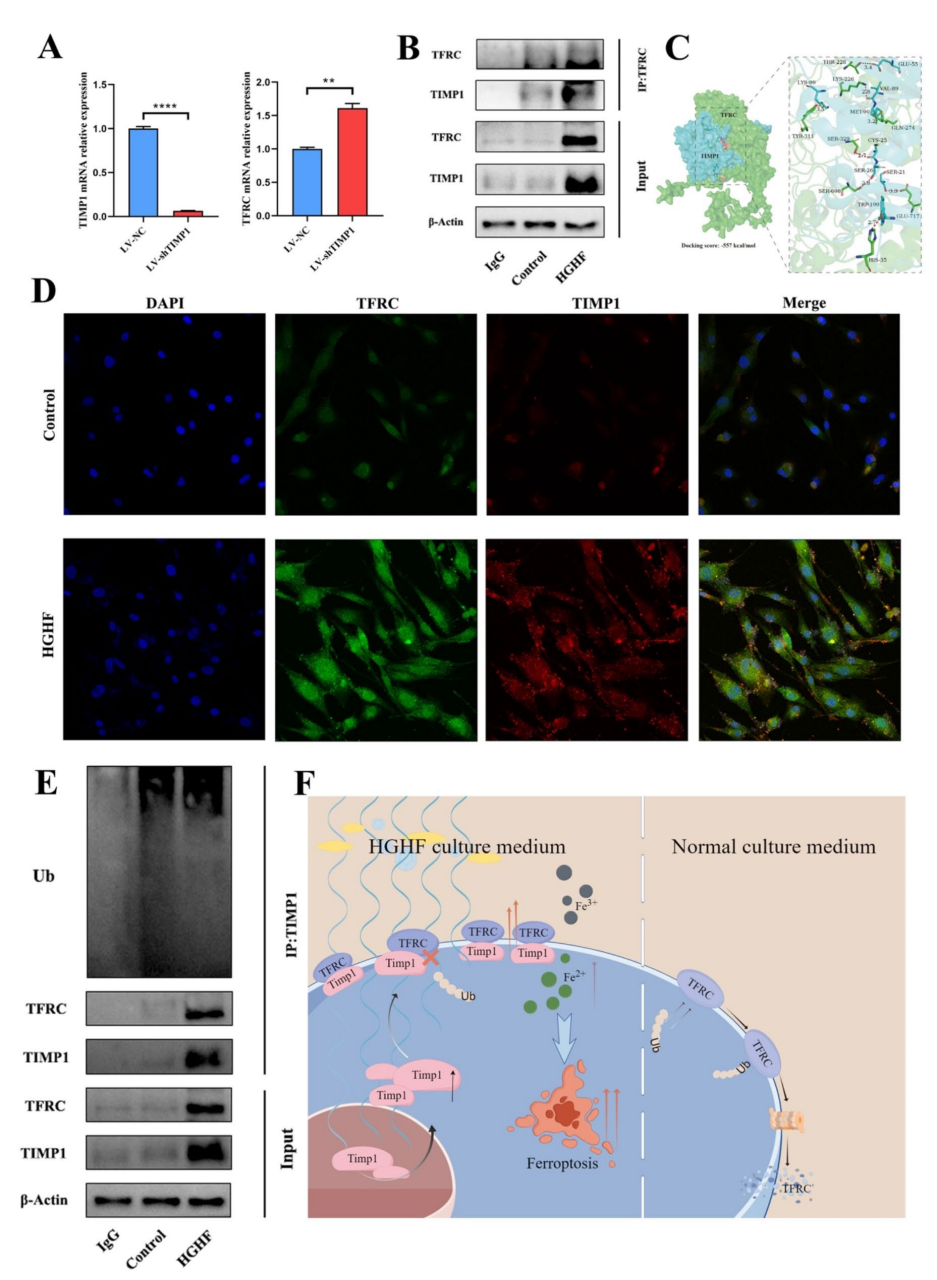
TIMP1 influences ferroptosis in osteoblasts by regulating the stability of TFRC protein. (**A**) qRT-PCR analysis of TIMP1 mRNA expression levels in LV-shTIMP1 and LV-NC groups. (**B**) Co-IP results showing immunoprecipitation of total cell lysates with protein A/G agarose beads (mock IgG) or anti-TFRC antibody in control and HGHF-treated osteoblasts, followed by immunoblotting with the indicated antibodies. (**C**) Schematic representation of TIMP1-TFRC molecular docking. (**D**) Two-photon laser confocal microscopy images showing immunofluorescence of TIMP1 (red) and TFRC (green) in control and HGHF-treated osteoblasts. (**E**) IP analysis of cell lysates using anti-TIMP1 antibody, followed by incubation with the indicated antibodies, including ubiquitination antibody, anti-TIMP1, and anti-TFRC, for WB analysis. (**F**) Schematic representation of the mechanism by which TIMP1-TFRC regulates osteoblast ferroptosis, leading to osteoporosis

To explore the interaction between TIMP1 and TFRC, we conducted a series of experiments. First, we measured TFRC mRNA expression levels in TIMP1-knockdown osteoblasts, finding that TFRC mRNA was significantly elevated in LV-shTIMP1 osteoblasts (Fig. 6A). Subsequently, endogenous co-immunoprecipitation (Co-IP) assays were performed to identify proteins interacting with TFRC, confirming a direct interaction between TIMP1 and TFRC. Notably, this interaction was present even in the absence of HGHF treatment (Fig. 6B). Rigid protein docking using GRAMM yielded 10 potential docking configurations, with the top-ranked conformation displaying the most favorable binding energy (-557 kcal/mol). This docking result was further analyzed using PyMOL to generate a 3D interaction model and determine hydrogen bond distances. The model showed that TFRC (yellow-green) and TIMP1 (cyan) form a stable complex, with hydrogen bonds including TYR311-LYS99 (3.5 Å), LYS226-VAL89 (2.8 Å), THR228-GLU55 (3.4 Å), GLN274-MET90 (3.2 Å), SER329-CYS25 (2.7 Å), SER698-SER26 (2.8 Å), GLU717-SER21 (3.3 Å), and HIS35-TRP199 (2.7 Å) (Fig. 6C). Immunofluorescence was performed in both Control and HGHF groups, with images captured using two-photon laser confocal microscopy. The HGHF group exhibited enhanced fluorescence intensities for both TIMP1 and TFRC, and co-localization of TFRC and TIMP1 at the cell membrane was observed in both groups (Fig. 6D). To further elucidate the relationship between these proteins, we conducted an additional endogenous Co-IP experiment to assess the ubiquitination levels of TIMP1-interacting proteins. The results confirmed the interaction between TIMP1 and TFRC, and demonstrated that in the HGHF group, the ubiquitination levels of proteins bound to TIMP1 were significantly reduced. Despite elevated protein levels of both TIMP1 and TFRC in the HGHF group compared to the Control group, their ubiquitination levels were not proportional (Fig. 6E). These findings suggest that TIMP1 binding to TFRC may attenuate TFRC ubiquitination, thereby modulating its role in ferroptosis.

### TIMP1 regulates osteoblast ferroptosis and diabetic osteoporosis

**Fig. 7:**
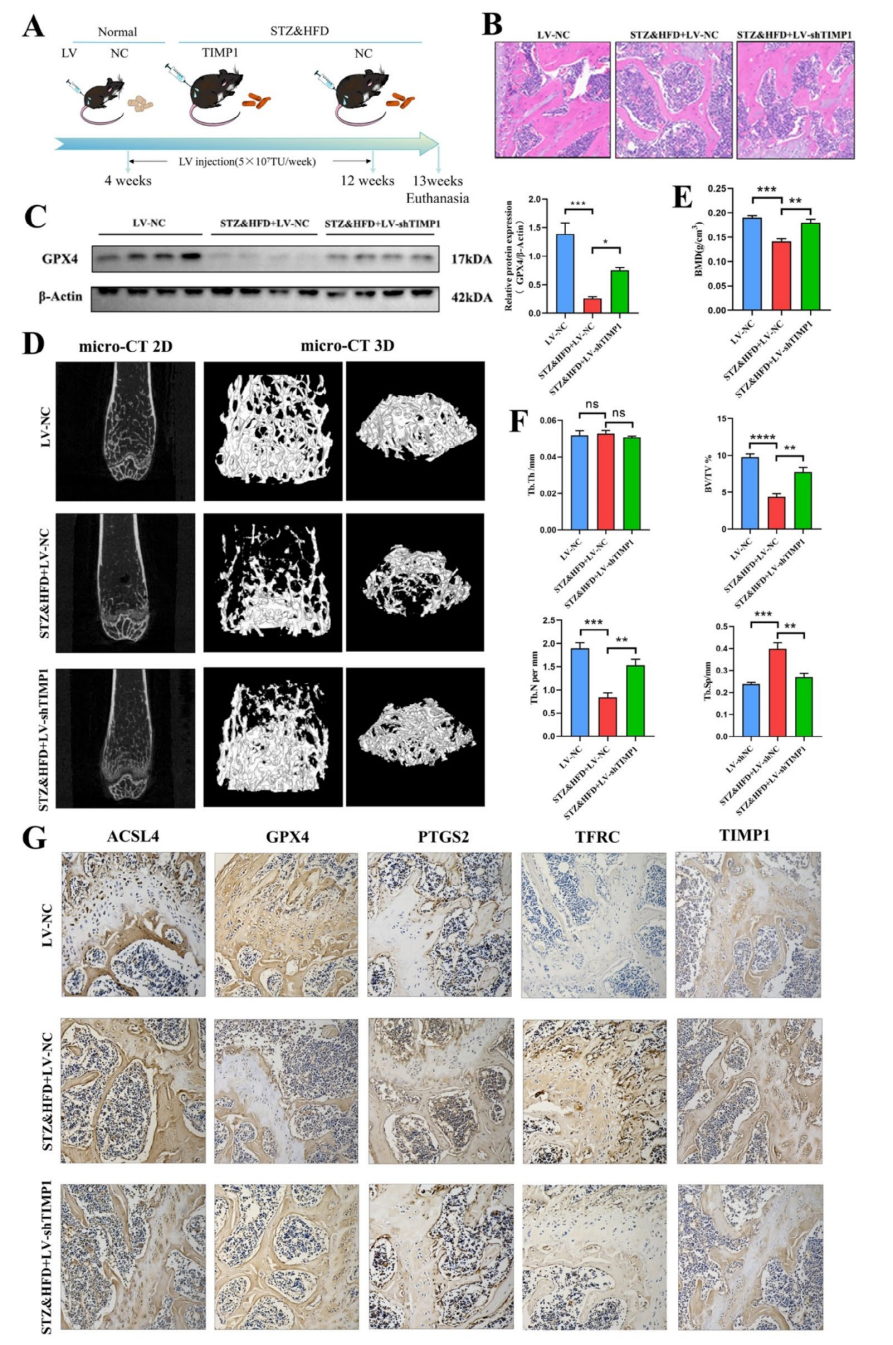
TIMP1 regulates osteoblast ferroptosis and diabetic osteoporosis. (**A**) Schematic representation of the grouping and lentiviral intervention in the type 2 diabetic mouse model. (**B**) Histological images of distal femoral tissue sections stained with H&E. (**C**) WB analysis of GPX4 expression levels in femoral bone tissue (*n* = 4), with semi-quantitative analysis based on WB results. (**D**) 2D and 3D micro-CT images of the distal femur in LV-NC, STZ&HFD + LV-NC, and STZ&HFD + LV-shTIMP1 groups of mice. (**E**) Quantitative analysis of trabecular bone density (BMD) at the distal femur in mice (*n* = 4). (**F**) Quantitative analysis of BV/TV, Tb.Th, Tb.Sp, and Tb.N in the trabecular bone of the distal femur in mice (*n* = 4). (**G**) Immunohistochemical images showing ACSL4, GPX4, PTGS2, TFRC, and TIMP1 expression in control and STZ&HFD mice (*n* = 4). Data are presented as mean ± SEM

To elucidate the role of TIMP1 in diabetic osteoporosis in vivo, we administered targeted femoral injections of LV-shTIMP1 (Fig. 7A). Comparative analysis between the STZ&HFD + LV-shTIMP1 and STZ&HFD + LV-NC groups revealed increases in trabecular bone surface area, trabecular number, and osteoblast count in representative H&E-stained images (Fig. 7B). Additionally, Micro-CT imaging demonstrated significant improvements in bone quality in the STZ&HFD + LV-shTIMP1 group compared to the other groups (Fig. 7D). Specifically, Micro-CT parameters showed a notable increase in bone mineral density (BMD), bone volume fraction (BV/TV), and trabecular number (Tb.N), coupled with a reduction in trabecular separation (Tb.Sp). Trabecular thickness (Tb.Th) remained unchanged (Fig. 7E, F). Immunohistochemical analysis indicated that TIMP1 knockdown significantly decreased the expression of TIMP1, ACSL4, TFRC, and PTGS2, while GPX4 expression was markedly upregulated (Fig. 7G). Western blotting further corroborated the substantial increase in GPX4 expression following TIMP1 knockdown (Fig. 7C). Collectively, these findings suggest that TIMP1 knockdown in vivo mitigates ferroptosis and ameliorates diabetic osteoporosis.

## Discussion

Extensive research has demonstrated that hyperglycemia and hyperlipidemia can directly impair osteoblast function (Xiao et al. 2022; Massera et al. 2018). However, the precise pathways involved in this process remain unclear, and the underlying mechanisms driving changes in bone tissue within the diabetic microenvironment are still not fully elucidated. In this study, we observed extensive osteoblast death in diabetic osteoporosis, accompanied by notable ferroptosis-related characteristics, including lipid peroxidation, increased ROS, Fe²⁺ accumulation, elevated MDA levels, and activation of ferroptosis-associated proteins. We further demonstrated that TIMP1 plays a critical role in osteoblast ferroptosis induced by the diabetic microenvironment, and this effect is mediated through its interaction with TFRC. Importantly, targeting TIMP1 significantly mitigated bone loss and osteoblast death. In this study, we successfully established a type 2 diabetes-induced osteoporosis mouse model by combining low-dose STZ injection with a high-fat diet (HFD). The STZ&HFD mice exhibited significant reductions in BMD, BV/TV, and Tb.N within the trabecular bone region, along with an increase in Tb.Sp. These findings are consistent with previous studies, suggesting that the diabetic microenvironment may primarily affect the most active regions of bone remodeling. Consequently, we focused our investigation on osteoblasts, which are predominantly located on the surface of trabecular bone and play a crucial role in bone remodeling.

In this study, we investigated the critical role of TIMP1-mediated deubiquitination of TFRC in ferroptosis of osteoblasts, focusing on its effects on Fe^2+^, ROS, and lipid peroxidation levels during the ferroptosis process. Iron dependence, a hallmark of ferroptosis, is characterized by increased labile iron pool (LIP) Fe^2+^, also known as unstable iron pool (Zhang et al. 2022a, b). While iron is essential for physiological processes, excessive iron can be detrimental, with elevated intracellular LIP playing a key role in ROS production through Fenton reactions and enzymatic activity. The production of ROS triggers lipid peroxidation, ultimately leading to ferroptosis. TFRC, as an essential iron transporter on the cell membrane, regulates the uptake of iron related to serum transferrin or lactoferrin, thereby influencing the occurrence of ferroptosis (Zuo et al. 2022; Lu et al. 2021). Under HGHF-induced conditions, our study explored the impact of TFRC on ferroptosis in osteoblasts. Our findings indicate that during HGHF induction, TFRC protein levels increase, accompanied by elevated Fe^2+^, ROS, lipid peroxidation, PTGS2 protein levels, and reduced GPX4 protein levels, all of which confirm the occurrence of ferroptosis. We hypothesize that TFRC may play a regulatory role in ferroptosis of osteoblasts. To test this hypothesis, we performed TFRC knockdown, which led to a suppression of ferroptosis in HGHF-stimulated osteoblasts. These results collectively demonstrate that TFRC plays a decisive role in ferroptosis of osteoblasts induced by HGHF stimulation.

Recent studies suggest that TIMP1 may be a potential biomarker and therapeutic target related to ferroptosis (Hou et al. 2023; Wang et al. 2024). However, the regulatory role of TIMP1 in ferroptosis appears to differ across various tissues and diseases (Gao et al. 2024; Sampilvanjil et al. 2020; Shi et al. 2022; Wang et al. 2023a, b). To our knowledge, this is the first study to investigate the mechanism of TIMP1 in diabetes Osteoporosis. We observed that TIMP1 is significantly elevated in osteoblasts following HGHF stimulation, which is associated with signs of ferroptosis. Osteoblasts with TIMP1 knockdown exhibited increased resistance to ferroptosis when exposed to HGHF. Specifically, these cells showed lower levels of Fe^2+^, ROS, MDA, and lipid peroxidation, while Western blot analysis indicated higher GPX4 and lower PTGS2 protein expression. These findings collectively suggest that TIMP1 plays a positive and active regulatory role in the ferroptosis of osteoblasts induced by HGHF.

Upon examining the impact of HGHF on TIMP1-knockout osteoblasts, we observed that TFRC protein levels were reduced in TIMP1-knockout osteoblasts compared to the Control group. Combining this with previous findings, we hypothesize that TIMP1 influences the ferroptosis process in osteoblasts by regulating TFRC. To validate this hypothesis, we overexpressed TFRC in TIMP1-knockout osteoblasts using a plasmid vector. We found that TFRC overexpression could reverse the enhanced resistance to ferroptosis caused by TIMP1 knockout, as evidenced by increased levels of Fe^2+^, ROS, and lipid peroxidation. Western blot analysis revealed that while the LV-shTIMP1 + oe-TFRC group had reduced TIMP1 protein levels, the GPX4 and PTGS2 protein levels showed results nearly opposite to those of the LV-shTIMP1 group. These results support the hypothesis that TFRC, as a downstream effector of TIMP1, directly influences ferroptosis in osteoblasts. Therefore, we can conclude that, in the HGHF microenvironment, TIMP1 directly affects ferroptosis in osteoblasts through its downstream effector TFRC.

In our investigation of the specific mechanism between TIMP1 and TFRC, RT-PCR results showed that the TFRC mRNA level was elevated in the LV-shTIMP1 group, indicating that TIMP1 knockout does not directly decrease TFRC transcriptional levels. This is consistent with our previous findings, which demonstrated a significant reduction in TFRC protein levels in the LV-shTIMP1 group. This suggests that TIMP1’s regulation of TFRC is more likely mediated at the protein level. An increase in mRNA levels is often a result of negative feedback regulation by low protein level (Shi et al. 2021), which is consistent with our experimental results. The results from immunofluorescence double staining indicate that TFRC and TIMP1 are co-localized outside the cell nucleus. This suggests an interaction between these two proteins within osteoblasts. Endogenous Co-IP experiments further confirmed the interaction between TIMP1 and TFRC. Additionally, molecular docking results support the notion that the interaction between TIMP1 and TFRC likely involves direct binding between the two proteins. TIMP1 is renowned not only for its inhibitory effects on matrix metalloproteinase activity but also for its role in regulating the ubiquitination process of other proteins through interactions within the cell, thereby affecting their stability.

Some studies have found that TIMP1 can attenuate ubiquitination modifications of target proteins through direct binding (Tang et al. 2024). Ubiquitination is a crucial protein degradation pathway that involves adding ubiquitin chains to proteins, marking them for degradation by the proteasome (Zhao et al. 2020; Liebelt et al. 2019; Ryu et al. 2022). In this study, endogenous Co-IP experiments assessed the ubiquitination levels of proteins interacting with TIMP1. The results confirmed that TIMP1 significantly reduces the ubiquitination of proteins bound to it. The stability of TFRC directly affects cellular iron uptake. If TIMP1 binds to TFRC and reduces its degradation, the number of active TFRC molecules on the cell surface will increase. This enhancement in TFRC availability will improve the cell’s ability to uptake transferrin-iron complexes, leading to increased intracellular iron content (Zhou et al. 2022; Xiahou and Han 2022; Liang et al. 2022). Observations from TFRC stability and Fe^2 +^ content in TIMP1 knockout and HGHF-treated cell models also support TIMP1’s positive effect on TFRC protein stability. Additionally, in vivo experiments indicated that TIMP1 knockout reduced TFRC levels in bone tissue while mitigating the effects of diabetic osteoporosis and promoting bone formation, consistent with previous in vitro findings. These results suggest that TIMP1’s influence on TFRC stability may be particularly significant under certain pathological conditions, where TIMP1 binding obstructs the effective action of the ubiquitination enzyme system, leading to reduced ubiquitination. The increased stability of TFRC enhances cellular uptake of Fe^2+^, resulting in elevated intracellular iron content. This iron accumulation, especially when excess Fe^2 +^ cannot be effectively utilized or removed, induces oxidative stress and lipid peroxidation, ultimately leading to cellular ferroptosis. Our study elucidates the role and mechanism of TIMP1 and its downstream effector TFRC in osteoblast ferroptosis within diabetic osteoporosis.

The study has several limitations. First, the investigation of ubiquitination mechanisms was not sufficiently in-depth. Second, the diabetes model used could not be completely controlled. Third, the study did not include diabetes treatment to observe changes in key genes and ferroptosis-related molecules. Lastly, in vivo knockout validation of the TFRC gene was not performed.

In conclusion, our research reveals a previously unknown mechanism where TIMP1, by binding to TFRC, inhibits ubiquitination and degradation, leading to increased TFRC stability and enhanced cellular sensitivity to ferroptosis. This finding not only uncovers a new role for TIMP1 in cellular iron homeostasis regulation but also provides new insights and potential therapeutic targets for understanding ferroptosis mechanisms and other iron metabolism-related diseases.

## Conclusion

TIMP1 expression is elevated in bone tissue in type 2 diabetes. In vitro, TIMP1 affects HGHF-induced ferroptosis in osteoblasts by regulating the stability of TFRC protein. In vivo, TIMP1 knockout reduces the severity of type 2 diabetes-induced osteoporosis. These findings suggest that TIMP1 is a potential therapeutic target for type 2 diabetic osteoporosis.

## Electronic supplementary material

Below is the link to the electronic supplementary material.

Supplementary Material 1

## Data Availability

The datasets utilized and/or analyzed during the present study are available from the corresponding author upon reasonable request.
