# Supplementary material for: TIMP1 regulates ferroptosis in osteoblasts by inhibiting TFRC ubiquitination: an in vitro and in vivo study
Source: Mol Med. 2024 Nov 23;30:226. doi: 10.1186/s10020-024-01000-9 (PMC11585138; doi:10.1186/s10020-024-01000-9)
Supplement: Supplementary file 1 — Supplementary Material 1 [file 10020_2024_1000_MOESM1_ESM.pdf]

LV-shTIMP1: Target Seg

Sh1-TIMP1: CCTGTTTATCTATCCCTTGCA
Sh2-TIMP1: CCTCTTGTTGCTATCACTGAT
Sh3-TIMP1: GGATTCAAGGCTGTGGGAAAT

Si-TFRC:

Si1-TFCR:
Sense Strand:GGGCUAUUGUAAGCGUGUATT
Antisense Strand: UACACGCUUACAAUAGCCCTT
Si2-TFCR:
Sense Strand:GGCGAGAUGAACACUAUGUTT
Antisense Strand: ACAUAGUGUUCAUCUCGCCTT
Si3-TFCR:
Sense Strand:GCCCAAGUAUUCUCAGAUATT
Antisense Strand: UAUCUGAGAAUACUUGGGCTT
Si4-TFCR:
Sense Strand:GCCCUAUGUAUCUCCAAGATT
Antisense Strand: UCUUGGAGAUACAUAGGGCTT

oe-TFRC:

ATGATGGATCAAGCCAGATCAGCATTCTCTAACTTGTTTGGTGGGGAACCATTGTCATA
CACCCGGTTTA
GCCTTGCTCGGCAAGTAGATGGAGATAACAGTCATGTGGAGATGAAACTGGCTGCAGA
TGAAGAAGAAAA
TGCCGACAATAACATGAAGGCTAGTGTCAGAAAACCCAAGAGGTTTAATGGAAGACTC
TGCTTTGCAGCT
ATTGCACTAGTCATTTTCTTCTTGATTGGATTCATGAGTGGCTACCTGGGCTATTGTAAG
CGTGTAGAAC
AAAAAGAGGAGTGTGTGAAACTGGCTGAAACGGAGGAGACAGACAAGTCAGAAACC
ATGGAAACAGAGGATGTTCCTACATCATCTCGCTTATATTGGGCAGACCTCAAAACACT
GTTGTCAGAGAAGTTGAACTCCATA
GAGTTTGCTGACACCATCAAGCAGCTGAGCCAGAATACATACACTCCTCGTGAGGCTG
GATCTCAAAAAG
ATGAAAGTCTTGCCTATTATATTGAAAATCAGTTCCATGAATTTAAATTCAGCAAAGTCT
GGCGAGATGA
ACACTATGTGAAGATTCAAGTGAAAAGCAGCATTGGTCAAAACATGGTGACCATAGTG
CAGTCAAATGGT
AACTTAGACCCAGTGGAGTCTCCCGAGGGTTATGTGGCATTCAGTAAACCTACAGAAG
TTTCTGGTAAAC
TGGTCCATGCTAATTTTGGCACTAAAAAGGACTTTGAAGAACTAAGTTATTCTGTGAAT
GGATCTTTAGT
GATTGTTAGAGCAGGGGAAATTACTTTTGCAGAAAAGGTTGCAAATGCCCAAAGCTTT

AATGCAATTGGT
GTCCTCATATACATGGACAAGAATAAATTCCCCGTTGTTGAGGCAGACCTTGCACTCTT
TGGACATGCTC
ATCTAGGAACTGGTGATCCATACACACCTGGCTTTCCTTCTTTCAATCATACTCAGTTTC
CGCCATCTCA
GTCATCAGGGTTGCCTAATATACCTGTGCAAACAATCTCAAGAGCTGCTGCAGAAAAG
CTATTTGGAAAA
ATGGAAGGAAGCTGTCCTGCTAGATGGAACATAGATTCTTCATGTAAGCTGGAACTTTC
ACAGAATCAAA
ATGTGAAGCTCATTGTGAAAAACGTACTGAAAGAAAGAAGAATACTTAACATCTTTGG
AGTTATTAAAGG
TTATGAGGAACCAGACCGTTATGTTGTAGTAGGAGCCCAGAGAGACGCTTTGGGTGCT
GGTGTTGCGGCG
AAGTCCAGTGTGGGAACAGGTCTTCTGTTGAAACTTGCCCAAGTATTCTCAGATATGAT
TTCAAAAGATG
GATTTAGACCCAGCAGAAGTATAATCTTTGCCAGCTGGACTGCAGGCGACTTTGGAGC
TGTTGGTGCCAC
TGAGTGGTTGGAGGGATACCTTTCATCTTTGCATTTAAAAGCTTTCACTTATATTAATTT
GGATAAAGTT
GTCCTTGGTACTAGTAACTTCAAAGTTTCTGCCAGCCCCTTATTATATACACTTATGGGA
AAGATAATGC
AAGATGTAAAGCATCCAGTTGATGGAAAATCTCTATATAGAGACAGCAATTGGATTAGC
AAAGTTGAGAA
ACTTTCCTTTGACAATGCTGCATATCCTTTCCTTGCATATTCTGGAATCCCAGCAGTTTC
TTTTTGTTTT
TGTGAGGATGCAGACTATCCTTATTTGGGCACTAGATTGGATACCTATGAGGCATTGACT
CAGAAAGTTC
CTCAGCTCAACCAAATGGTTCGTACAGCAGCGGAAGTGGCTGGTCAGCTCATTATTAA
